# 

**Published:** 1896-05-02

**Authors:** 


					Tffn HOSPITAL. ABSOLUTELY PURE, therefore BEST*
May 2nd, 1896.
CADBURY'S ^ ww ^ CADBURY'S
" Th,? standard ?f highest purity at present "==* lJMCr 1 NO ALKALIES USED,
attainable.?Lcuutt,
No. 50i>i Vol. xx. WITH EXTRA NUR8IN6 8UPPLENIENT. Pj^jwgraw?.
a .,,   ^ *r 1 one pieffistered at the General Post Oflloe aa a Newspaper for Monthly Parts, 6d.; post free, 8d
uatukDAY, JM.AY ZND, loyo, transmission in the United Kingdom and Abroad.] Ditto per Annum, 8s.6d., post free.

				

